# Health practitioner regulation and national health goals

**DOI:** 10.2471/BLT.21.287728

**Published:** 2023-06-29

**Authors:** Agya Mahat, Ibadat S Dhillon, David C Benton, Martin Fletcher, Francis Wafula

**Affiliations:** a Health Workforce Department, World Health Organization, Avenue Appia 20, 1202 Geneva, Switzerland.; b Department of UHC/Health Systems, World Health Organization Regional Office for South-East Asia, New Delhi, India.; c National Council of State Boards of Nursing, Chicago, Illinois, United States of America.; d Australian Health Practitioner Regulation Agency, Melbourne, Australia.; e Institute of Healthcare Management, Strathmore University, Nairobi, Kenya.

## Abstract

The role of health practitioner regulation in ensuring patient safety is well recognized. Less recognized is the role of regulation in addressing broader health system priorities. These goals include managing the costs, capacities and distribution of health professional education institutions; ensuring the competence and equitable distribution of health workers; informing workforce planning and mobilization; enabling the use of digital technologies; and addressing challenges related to the international mobility of health workers. Even where health practitioner regulation is designed to advance these goals, important gaps exist between the potential of regulatory systems and their performance. The response to the coronavirus disease 2019 (COVID-19) pandemic led many countries to introduce regulatory changes to allow more flexibility and innovations in the mobilization of health practitioners. Building on this experience, we need to critically re-examine health practitioner regulatory systems to ensure that these systems support rather than impede progress towards national health goals. We discuss the role of health practitioner regulation in contemporary health systems, highlighting recent regulatory reforms in selected countries, including during the COVID-19 pandemic. We identify the importance of dynamic, effective and flexible health practitioner regulatory systems in progress towards universal health coverage and health security.

## Résumé

Le rôle qu'exerce la réglementation relative aux professionnels de la santé pour la sécurité du patient est bien connu. En revanche, celui qu'elle joue dans la définition des grandes priorités du système de santé l'est moins. Elle comporte ainsi plusieurs objectifs, notamment: assurer la gestion des coûts, des capacités et de la distribution des établissements de formation des professionnels de la santé; garantir une répartition équitable d'agents de santé compétents; guider la planification et la mobilisation de la main-d'œuvre; permettre l'intégration des technologies numériques; et enfin, relever les défis liés à la mobilité internationale des acteurs du secteur. Même dans les endroits où une telle réglementation est conçue pour atteindre ces objectifs, des écarts considérables subsistent entre le potentiel des systèmes de réglementation et leurs performances réelles. La lutte contre la pandémie de maladie à coronavirus 2019 (COVID-19) a encouragé de nombreux pays à modifier les règles en vigueur en vue d'offrir une plus grande flexibilité et davantage d'innovations en matière de mobilisation des agents de santé. En nous fondant sur cette expérience, nous devons impérativement réexaminer les systèmes de réglementation propres aux praticiens afin qu'ils constituent un moteur, et non un frein, à la poursuite des objectifs nationaux en matière de santé. Dans le présent document, nous évoquons le rôle de la réglementation relative aux professionnels de la santé dans les systèmes de santé actuels, en mentionnant les récentes réformes entreprises par une série de pays dans ce domaine, y compris durant la pandémie de COVID-19. Nous soulignons également l'importance d'instaurer des systèmes de réglementation dynamiques, efficaces et flexibles pour les praticiens sur la voie menant à la sécurité sanitaire et à une couverture sanitaire universelle.

## Resumen

La función de la reglamentación de los profesionales sanitarios para garantizar la seguridad de los pacientes es bien conocida. Sin embargo, se reconoce menos la función de la reglamentación para atender prioridades más amplias del sistema sanitario. Estos objetivos incluyen: gestionar los costes, las capacidades y la distribución de las instituciones de formación de profesionales sanitarios; asegurar la competencia y la distribución equitativa de los trabajadores sanitarios; informar la planificación y movilización del personal; permitir el uso de tecnologías digitales; y abordar los retos relacionados con la movilidad internacional de los trabajadores sanitarios. Incluso en los casos en que la reglamentación de los profesionales sanitarios está concebida para promover estos objetivos, existen diferencias considerables entre el potencial de los sistemas de reglamentación y sus resultados. La respuesta a la pandemia de enfermedad por coronavirus de 2019 (COVID-19) llevó a muchos países a introducir cambios en la reglamentación para permitir una mayor flexibilidad e innovaciones en la movilización de los profesionales sanitarios. A partir de esta experiencia, es necesario volver a examinar de forma crítica los sistemas de reglamentación de los profesionales sanitarios para garantizar que estos sistemas apoyen y no impidan el progreso hacia los objetivos sanitarios nacionales. En este artículo se analiza la función de la reglamentación de los profesionales de la salud en los sistemas sanitarios contemporáneos y se destacan las recientes reformas reglamentarias introducidas en algunos países, en particular durante la pandemia de COVID-19. Asimismo, se destaca la importancia de que los sistemas de reglamentación de los profesionales sanitarios sean dinámicos, eficaces y flexibles para avanzar hacia la cobertura sanitaria universal y la seguridad sanitaria.

## ملخص

يحظى الدور الذي يلعبه تنظيم الممارسين الصحيين في ضمان سلامة المرضى، بتقدير جيد للغاية. ويحظى بتقدير أقل دور التنظيم في معالجة أولويات النظام الصحي الأوسع نطاقاً. وتشمل هذه الأهداف: إدارة التكاليف والقدرات وتوزيع مؤسسات التعليم المهني الصحي؛ وضمان الكفاءة والتوزيع العادل للعاملين في المجال الصحي؛ وإعلام تخطيط القوى العاملة وحشدها؛ وتمكين استخدام التقنيات الرقمية؛ والتصدي للتحديات المتعلقة بالحشد الدولي للعاملين في المجال الصحي. حتى عندما يكون تنظيم الممارسين الصحيين مصممًا للارتقاء بهذه الأهداف، توجد فجوات مهمة بين إمكانات الأنظمة التنظيمية وأدائها. أدت الاستجابة لجائحة مرض فيروس كورونا 2019 (كوفيد 19) إلى قيام العديد من الدول بإدخال تغييرات تنظيمية للسماح بمزيد من المرونة والابتكارات في حشد الممارسين الصحيين. بناءً على هذه التجربة، نحن نحتاج إلى إعادة فحص النظم التنظيمية للممارسين الصحيين بشكل دقيق للتأكد من أن هذه الأنظمة تدعم التقدم نحو الأهداف الصحية الوطنية، ولا تؤدي لإعاقته. نحن نناقش دور تنظيم الممارسين الصحيين في النظم الصحية المعاصرة، مع تسليط الضوء على الإصلاحات التنظيمية الأخيرة في دول محددة، بما في ذلك أثناء جائحة كوفيد 19. نحن نحدد أهمية النظم التنظيمية الديناميكية والفعالة والمرنة للممارسين الصحيين في التقدم نحو التغطية الصحية الشاملة والأمن الصحي.

## 摘要

实施卫生从业人员监管的作用是确保卫生从业人员高度重视患者的安全。监管在处理更广泛的卫生系统优先事项方面所发挥的作用普遍未得到重视。这些目标包括：管理卫生专业人士教育机构的费用、能力和分布；确保卫生工作者具备一定能力和得到公平的分配；为劳动力规划和动员提供信息；促进数字科技的使用；以及应对与卫生工作者的国际流动相关的挑战。即使是在专用于推进实现这些目标的卫生从业人员监管工作方面，监管制度的潜力与其绩效之间也存在着重大差距。为应对 2019 年新型冠状病毒肺炎 (COVID-19) 疫情，许多国家进行了监管改革，以便在动员卫生从业人员方面保持更高的灵活性和创新性。在这一经验的基础上，我们需要严格地重新审查卫生从业人员监管制度，以确保这些制度支持而不是阻碍国家卫生目标的进展。我们讨论了卫生从业人员监管在当代卫生体系中的作用，重点介绍了一些国家最近推行的监管改革，包括在 2019 年新型冠状病毒肺炎疫情期间的监管改革。我们认识到动态、有效和灵活的卫生从业人员监管制度在实现全民健康覆盖和促进卫生安全中发挥重要作用。

## Резюме

Роль регулирования деятельности практикующих медицинских работников в обеспечении безопасности пациентов является общепризнанной. Менее признанной является роль регулирования в отношении более обширных приоритетов в системе здравоохранения. В числе этих целей: управление расходами, возможностями и распределением учреждений профессионального образования в области здравоохранения; обеспечение компетентности и равноправного распределения медицинских работников; информационное обеспечение планирования и мобилизации рабочей силы; создание условий для использования цифровых технологий; решение проблем, связанных со свободой трудоустройства иностранных работников в сфере здравоохранения. Даже там, где регулирование деятельности практикующих медицинских работников направлено на достижение этих целей, существуют значительные разрывы между потенциалом систем регулирования и их эффективностью. Ответные меры на пандемию коронавирусной болезни 2019 г. (COVID-19) заставили многие страны внести изменения в нормативно-правовую базу с целью обеспечения большей гибкости и инноваций в мобилизации практикующих медицинских работников. Опираясь на этот опыт, необходимо тщательно пересмотреть системы регулирования деятельности практикующих медицинских работников, чтобы убедиться, что эти системы поддерживают прогресс в достижении целей здравоохранения на национальном уровне, а не препятствуют ему. Обсуждается роль регулирования деятельности практикующих медицинских работников в современных системах здравоохранения, освещаются недавние реформы регулирования в отдельных странах, в том числе во время пандемии COVID-19. Поэтому в процессе продвижения к всеобщему охвату услугами здравоохранения и безопасности здоровья особое внимание уделяется динамичным, эффективным и гибким системам регулирования деятельности практикующих медицинских работников.

## Introduction

Occupational regulation has been defined as “the legally defined requirements or rules that govern entry into occupations and subsequent conduct within them.”^1^ Systems for regulation of health practitioners are designed to address the asymmetry in knowledge between patients and practitioners, and reduce the risk of harm to patients. Regulations covering health practitioners generally include:^2^ (i) educational requirements for professional practice through the establishment of education standards and quality assurance of education programmes; (ii) a system of registration or licensure which may include establishment of professional codes of conduct, identification of protected titles or regulated scopes of practice and requirements for maintenance of registration; (iii) a register of those who are licensed or registered, which may be accessible to the public and include any special requirements or restrictions on registration or licensure; and (iv) processes for dealing with concerns about regulated practitioners, and systems for implementing appropriate disciplinary measures in cases of professional misconduct, sub-standard performance or addressing impairment in physical or mental capacity.

The systems for health practitioner regulation are under increasing pressure in many countries due to several factors: the growth and privatization of health professional education; the increasing prominence of previously unregulated occupations, and the emergence of new occupations and new technologies in health care; emergencies and humanitarian crises; increasing international mobility of practitioners; a growing focus on team-based and integrated networks for service delivery; and increasing consumer demand, expectations and knowledge.^3^

The coronavirus disease 2019 (COVID-19) pandemic has further highlighted the need for health practitioner regulation to be more readily updated, with room for flexibility and better alignment to current national health priorities. Across countries, the adaptations made to health practitioner regulation were fundamental to ensuring the rapid availability of health practitioners for the national emergency response. The lessons of the pandemic create an opportunity to examine the role of regulation in advancing health system needs and priorities, and to consider how innovations and greater flexibility in regulation can be applied without compromising patient safety.^4^

Health practitioner regulation is increasingly recognized as a core mechanism to ensure the availability, accessibility, acceptability and quality of the health workforce.^5^^,^^6^ Through an overview of diverse health practitioner regulatory systems, we highlight the evolving role of health practitioner regulation in contemporary health systems, including in low- and middle-income countries. We describe recent regulatory reforms and the adaptions during the COVID-19 pandemic, and discuss the opportunity to strengthen the alignment of health practitioner regulations with national health goals.

## Diversity of systems

Health practitioner regulation encompasses multiple aims, with substantial diversity in the structure and operation of regulations across countries with different geography, economy and socio-political history.^2^ Examples include self-regulation (regulation administered exclusively by individuals who are members of the professions being regulated); independent statutory authority (an authority established by law specifically for regulatory functions); co-regulation (delegation of certain regulatory functions to the professional associations); direct government regulation (regulation administered by government entities); and combinations of the above. The regulatory approaches can range across competencies (assessing individual practitioners); risk of harm (assessing risk of practitioner intervention or activity); scope of practice (limiting roles, activities or authority of practitioner or occupations); and controlled acts (limiting specific procedures or tasks to authorized practitioners or occupations).^7^

The health occupations that are regulated and the type of regulation can vary across countries and jurisdictions. Regulation may also be non-statutory, as in the case of voluntary regulation. There may even be no regulation for certain health occupations in some cases, except for generic rules that are applicable to all occupations in a country. Depending on the type of health occupation and the country, regulation may differ across sub-national jurisdictions, and regulatory functions may be separated across different authorities.

**Fig. 1:**
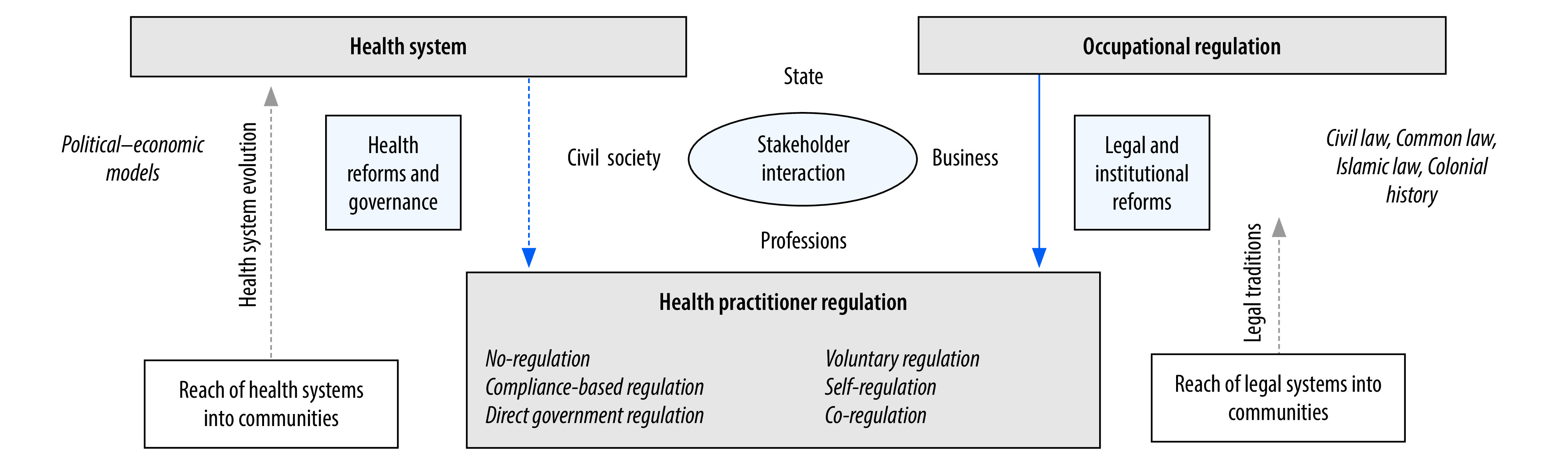
Determinants for design of health practitioner regulatory systems

Note: Health system and occupational regulation are the major elements that determine the design of health practitioner regulation in a jurisdiction. The influence of occupational regulation (blue solid arrow) is more than the health system (blue dashed arrow) on health practitioner regulation even though the latter reaches deeper into communities. These reaches (grey dashed arrows) are, in turn, determined by contextual factors such as political and economic models, colonial history, and legal traditions. The balance of power between the state, professions, civil society and businesses, also influence health practitioner regulation. As a result, there is substantial diversity in how health practitioners are regulated across and within countries.

The diversity in health practitioner regulatory systems can be explained by the context in which they are established and operate (Fig. 1). Systems for occupational regulation are influenced by the legal tradition, political economy and colonial history of a country. At the centre of the system are the interactions among government, business, professions and civil society. Historically, health professions in anglophone countries have followed the British model of self-regulation, while in other political systems, governments have more control over health practitioner regulation. The design of health practitioner regulation often follows occupational regulation models in the country, rather than the health system in which the health practitioners work. In fact, health practitioner regulation may be disconnected from or lag behind health system reforms.^8^ However, health systems tend to have a greater reach into communities than legal systems, and pragmatic local norms can override formal regulatory provisions for health practitioners in remote and underserved geographical locations.

## Low- and middle-income countries

The capacity of regulators to implement regulatory policies and perform their functions is fundamental for health practitioner regulation to be effective. There is concern, however, that the establishment of health practitioner rules and statutes in low- and middle-income countries may outpace a country’s ability to implement them. 

Despite established regulatory systems, wide variation exists in the quality of practitioners within and across low- and middle-income countries. These variations raise questions over the standardization of qualifications, job titles and minimum competency, and point to concerns over the sometimes low quality of health practitioners, including regulated professions.^9^^,^^10^

Health practitioner registries provide information on regulated health practitioners and are the most common source of data on the health workforce. However, enforcement of registration requirements and registry maintenance has been a major challenge. Studies show that in some countries only 44% of physicians working in the public sector were registered,^11^ and in others only half of the registered practitioners were in active practice (10 898 out of 21 731 health practitioners in some areas, and 7719 out of 17 443 health practitioners in others).^12^ In countries with federated systems, health workforce data recorded by the professional councils may not account for migration, death or double-counting of practitioners who are registered in more than one state.^13^

Operational challenges may worsen the situation. In some settings, delays in procurement of certificates have led to long suspensions in issuing licenses,^14^ including during the COVID-19 pandemic. Financial resources are a key barrier to strengthening regulatory systems in low- and middle-income countries. For instance, in 2011, the administrative cost of Kenya’s regulatory system on health practitioners was estimated to be 13.2 million United States dollars, equivalent to around 0.6% of the country’s total health expenditure.^15^

Enforcement of regulations for short-term international health practitioners is also a challenge in low- and middle-income countries. Anecdotal evidence suggests that international medical volunteers and emergency health workers may at times perform tasks beyond their expertise or training, disregarding local requirements and escaping liability for undertaking procedures that would subject them to regulatory action in their home jurisdiction.^16^^–^^18^

The limited effectiveness of regulators in addressing professional and ethical misconduct of practitioners is also a concern in some low- and middle-income countries. Challenges include lack of public awareness on their entitlement to services and complaint mechanisms; power imbalances between health practitioners and the public; inadequate alternatives to health practitioners; and weak mechanisms for addressing medical negligence and practitioner misconduct.^19^^–^^22^

In some low- and middle-income countries, the strong influence of their colonial history on the objectives and approach to health practitioner regulation has contributed to the current regulatory challenges faced. Often health practitioner regulatory systems were established by the former colonial governments following the model in their respective country of origin, with little consideration given to the local context and population needs.^11^^,^^23^^,^^24^ Even after independence, these systems were rarely reformed according to evolving population and health system priorities, in contrast to the periodic reforms in high-income countries.

## Recent reforms

Despite the diversity of health practitioner regulatory systems, the core purpose of these systems is to serve the public. However, the definition of public interest is itself contested, as it varies across jurisdictions and has evolved over time, primarily through assuring competency and minimum standards of health practitioners. While an earlier interpretation of the public interest was viewed as synonymous with elevating the status of professions, this understanding is increasingly shifting towards defining value from the perspective of communities. Public interest is now seen as prioritizing the efficiency, value-for-money, quality and safety of health services, and the responsiveness of the regulatory system to the complex and evolving needs of health systems. Public interest also includes whether the regulatory intervention is proportional to the risks presented by the practitioner and to the benefits accrued from the intervention and the regulatory burden imposed.^25^ Box 1 compares the contemporary understanding of goals in the public interest of health practitioner regulation with that in the 19th and 20th centuries.

Box 1 **Evolving public-interest goals of health practitioner regulatory system design**

**19th to mid-20th century**

• Standards of practice

• Standards of qualification

• Elevating the profession

• Addressing public information deficit

• Entry barriers

• Competence of practitioner

• Access to services

**21st century**

• Costs of regulation

• Increased efficiency

• Increased cost–effectiveness

• Reduction in entry barriers to the profession

• Reduction of barriers to professional mobility

• Promoting competition

• Regulation proportionate to risk

• Promoting alternatives to the licensure model

• Responsive to a highly complex health system

• Uniformity in regulations

• Alignment with health system needs

Source: Adapted from Benton et al. 2019.^25^

Table 1 highlights recent reforms in six countries that had traditionally followed a model of self-regulation. These reforms illustrate how the rationale for health practitioner regulation is expanding beyond patient safety to broader purposes, such as health workforce sustainability, promotion of wider health-sector goals and universal health coverage (UHC).

**Table 1:** Contemporary reforms in health practitioner regulation in selected countries

Country	Act	Objective	Key features
Australia	Health Practitioner Regulation National Law Act of 2009; Amendment 2019^5^	• Provide public protection. • Facilitate workforce mobility. • Facilitate high-quality education and assessment of foreign-trained practitioners. • Facilitate patients’ access to services. • Enable development of a flexible and sustainable health workforce.	• The Australian Health Practitioner Regulation Agency and 15 profession-specific national boards replaced the individual state regulatory bodies for different health professions. • A government-appointed management committee exists that includes non-health practitioners, and those with expertise in health, education, business, or administration. • Members of the national boards are appointed by the government and comprise practitioners (50%–66%) and community members from each jurisdiction, the profession being regulated, and region or rural area. • Oversight is provided by the council of health ministers of each state or territory and the Commonwealth of Australia. • The Agency maintains a single national register of all regulated health practitioners which is publicly available online. • For unregulated health professions, the professional associations provide guidance on standards and the national code of conduct sets minimum standards for non-registered health-care workers.
India	National Medical Commission Act of 2019^6^	• Provide a medical education system that improves access to quality and affordable medical education, and ensures the availability of adequate and high-quality medical professionals in all parts of the country. • Promote equitable and universal health care that encourages a community health perspective and ensures all citizens can access services.	• The National Medical Commission replaced the regulatory function of the Medical Council of India. • The Commission comprises a medical advisory council and four autonomous boards. • The members include a government-appointed chairperson; representatives of the government agencies, autonomous boards, academic and research institutions; and part-time members that include representatives from the state medical councils. • A national entrance examination for admission into undergraduate medical programmes, and a common undergraduate final year national exit examination for registration or licensure to practice and for entry into postgraduate programmes made mandatory. • Limited prescription and preventive care licensure granted to community health providers. • The Commission determines the fees and other charges of 50% of the seats in private medical colleges.
Kenya	The Health Act No. 21 of 2017^26^	• Establish a unified health system. • Coordinate the relationship between national government and county government health systems. • Provide for regulation of health-care services and providers, health products and health technologies.	• The Kenya Health Professions Oversight Authority established to coordinate and promote inter-professional liaison among the regulatory bodies. • The board of the Authority comprises representatives from the government, health professions, private sector and the public and is chaired by an appointed health professional. • The Authority maintains a duplicate register of all health professionals and addresses complaints and grievances. • The Authority monitors implementation of the functions of regulatory bodies, arbitrates disputes and ensures standards for health professionals.
Nepal	National Medical Education Act of 2075^27^	• Increase state investment in medical education. • Regulate medical education in an integrated and efficient manner. • Develop medical education in alignment with national needs. • Ensure equal access of all students including students from deprived backgrounds.	• The Nepal Medical Education Commission was established to regulate the education sector of health professions and improve its quality, replacing the function of the professional councils on education and accreditation. • The Commission is chaired by the prime minister, while the ministers for education and health are co-chairs; the vice-chair is appointed by the government. Other members include representatives from the government, academics, professional councils, professional associations, civil society, and private medical and dental colleges. • Ten years after the adoption of the National Medical Education Act, all medical education institutions will start to be not-for-profit institutions. • At least 75% of the seats in public institutions and 10%–20% of the seats in private institutions should be allotted to scholarship students, and everyone on government scholarship will have to sign a bond to work in government-deployed areas for 2 years or pay a financial penalty. • The Commission sets a limit on the education cost charged to students in the private sector, grants accreditation to education institutions and conducts a common entrance examination for entry into undergraduate and postgraduate programmes. • No new medical, dental or nursing college can be established in the Kathmandu valley for 10 years after the adoption of the Act.
New Zealand	Health Practitioners Competence Assurance Act of 2003^28^	• Protect the health and safety of the public by providing mechanisms to ensure that health practitioners are competent and fit to practise their professions.	• The 11 regulatory authorities are responsible for regulation of existing regulated health professions. It is possible for regulatory authorities to be merged and to add new health professions to be regulated at the recommendation of the health ministry. • Each authority comprises a maximum of 14 members, most of whom are elected health practitioners, but the authority includes at least two to three lay members. • Among others, the functions of each regulatory authority include: specifying the qualifications required for their scope of practice within the profession; authorizing registration of health practitioners and maintaining registers; notifying employers and other relevant authorities if the practise of health practitioners may pose a risk of harm to the public; liaising with other regulatory authorities about matters of common interest; promoting and facilitating interdisciplinary collaboration and co-operation; and promoting public awareness of the responsibilities of the authority. • A performance review of each authority must be conducted at least every 5 years through an independent person appointed by the health ministry. • Regulated health practitioners can perform health services beyond the scope of practice during emergencies.
United Kingdom	NHS Reform and Health Professions Act of 2002 (latest available version)^29^	The objective of the Professional Standards Authority for Health and Social Care is to: • Protect, promote and maintain the health, safety and well-being of the public. • Promote and maintain public confidence in the professions regulated by the regulatory bodies. • Promote and maintain proper professional standards and conduct for members of those professions.	• The Professional Standards Authority for Health and Social Care provides oversight to the professional councils as a co-regulator and is accountable to the parliament. • The Authority’s board comprises professionals who are not regulated by the Act. The professional councils are not represented in the Authority’s board. • The Authority reviews decisions made by the nine regulators about practitioners’ fitness to practise. • The Authority can accredit voluntary registers that meet the standards and suspend accreditation, apply conditions and remove accreditation. • Elections in the professional councils are replaced with members appointed by the government, with 50% representation from lay members. • The General Medical Council established a medical practitioners tribunal service to separate the investigation and adjudication functions.

NHS: National Health Service.

The objective of Australia’s national registration and accreditation scheme includes ensuring “workforce mobility across Australia” and enabling the “continuous development of a flexible, responsive and sustainable Australian health workforce.”^5^ India’s National Medical Commission replaced the Medical Council of India that was established during the British colonial period. The National Medical Commission Act aims to “ensure availability of adequate and high-quality medical professionals in all parts of the country,” “promote equitable and universal health care that encourages community health perspective,” and “promote national health goals.”^6^ Nepal’s Medical Education Commission regulates all health professional education institutions and programmes, which used to be the responsibility of various professional councils and government entities. The law on medical education is intended to develop professional education “in alignment with national needs,” and “ensure equal access to all students including the deprived.”^27^ New Zealand’s health practitioners’ competency assurance system includes elements to “promote and facilitate interdisciplinary collaboration and cooperation in the delivery of health services,” and “to promote public awareness of the responsibility of authorities.”^28^

Common themes related to reform in these six countries in Table 1 include improving transparency about the appointment of regulators; how they operate; and their regulatory decision-making and social accountability. The role of communities in regulation is also growing, with greater representation of lay members on regulatory boards.

Efforts to balance power across the state, the professions and civil society include establishing oversight bodies for regulatory authorities in Kenya and the United Kingdom of Great Britain and Northern Ireland; replacing elected members of the profession within the regulatory boards with appointed members; and including public representatives on regulatory boards in Australia, India, Nepal and the United Kingdom.

Recent reforms have been triggered by a variety of factors. In some countries the public outcry over risk to patient safety from regulated professions,^30^ and regulatory inefficiencies and corruption have led to reforms.^31^ In other countries, reform was prompted by substantial growth of the private sector; along with increased demand for quality care and increased influence of international standards;^32^^,^^33^ concerns over cost and quality of medical education in the private sector;^34^ and the need to ensure that future national health workforce requirements can be met.^5^

The patterns of reform are not uniform, however. In several anglophone countries, regulatory models tend to be shifting from self-regulation to systems with stronger state oversight.^5^^,^^6^^,^^9^ However, countries with traditionally stronger government involvement, such as China and Republic of Korea, are delegating certain regulatory functions to the professions.^35^^,^^36^ Indonesia is considering a draft Health Omnibus law that brings together health practitioner regulation and other health laws in support of national health priorities.^37^

Reforms in Australia created uniform national standards for regulated health practitioners, and a single agency responsible for regulatory functions.^5^ However, implementation of national standards can be challenging in contexts with substantial sub-national variations in health and other socioeconomic conditions.^10^ Major reforms may not endure when there is a lack of consensus among stakeholders, or political instability.^38^

A cross-country historical review of health practitioner regulatory reforms over the last century further suggests that successful reforms, at minimum, require responsiveness to the local context; a clear understanding of regulatory goals to be addressed; and collaboration across relevant stakeholders (such as government, the public, professions, employers and businesses).^39^

## Regulation in health emergencies

Emergencies such as disasters, humanitarian crises and disease outbreaks place enormous stress on regulatory systems due to the heightened demand for qualified health practitioners. In such situations, regulatory flexibility enables the rapid deployment of additional health practitioners to respond to the emergency and to maintain essential health services. Box 2 presents selected examples of information on regulatory adaptations applied in different countries during the COVID-19 pandemic.

Box 2 **Examples of emergency flexibilities and adaptations in health practitioner regulation in selected countries**

**Entry to practice**

- Deployment of health professional students: France, India
- Early or expedited graduation of health professional students: Italy, USA
- Waivers on registration or licenses or validity of out-of-state license: Canada, Peru, USA
- Waivers on registration or license renewal: Netherlands (Kingdom of the), South Africa, USA
- Re-employment of retired practitioners: Australia, New Zealand, Spain, USA

**Scope of practice**

- Expansion of scope of practice: Ireland, New Zealand, USA

**Telemedicine**

- Waivers on registration requirements in domicile of patients and practitioners: USA
- Permission to use telemedicine without prior in-person consultation: Indonesia, South Africa, New Zealand
- Issuing of guidelines and permits to practise specific telemedicine services: Colombia, Greece, India, Ireland, Nepal, Philippines
- Expanding the scope of telemedicine services or specifying what services can be provided: France, Peru

**International mobility**

- Reinstatement of expired licenses: Brazil
- Waivers on validity of foreign licenses and licensing examinations: Chile, Spain, USA (New York State), USA (New Mexico State)
- Waivers on validation of qualifications: European Union, Peru
- Supervised clinical practice for unregistered practitioners or limited permits: United Kingdom of Great Britain and Northern Ireland, USA

Examples of flexibility on regulatory requirements for entry into practice include: early or expedited graduation of health practitioners in the United Kingdom; temporary waivers on requirements for registration and licensing for new graduates and out-of-state practitioners in the United States of America (USA); waivers on renewals of licensure or automatic renewals of past registrants in South Africa; and allowing mobilization of students to clinics in India. In the USA, the scope of practice of existing health practitioners was expanded to allow health practitioners to work to the full capability of their education or training, and therefore enable the available human resources to be repurposed. The use of telemedicine was also encouraged by regulators in several settings. Examples include relaxations in licensing requirements in the USA; regulatory amendments to allow telemedicine services without a prior in-person consultation in South Africa; and the issuing of guidance on telemedicine in Nepal. Examples of relaxations in regulations to encourage entry and utilization of international health practitioners include instituting waivers on the validation of qualifications in Peru; allowing waivers on licensure examinations or granting validity to foreign licensure in Chile; reinstating the licensure of international practitioners in Brazil; and permitting unregistered international practitioners to perform clinical tasks under supervision in the United Kingdom.

The pandemic also highlighted the role of regulatory bodies in supporting workforce planning. While many countries struggled with collating data on their active health workforce, Australia’s regulatory authority was able to swiftly reinstate the registration of almost 40 000 practitioners who had left practice, without any application forms or fees, to support the government in planning for the surge response.^40^

While most of the regulatory flexibility was temporary and enabled through executive orders, some countries have made long-term changes from lessons learned during the pandemic. These changes include developing legislation for regulating telemedicine services,^41^ and in some cases permanently abolishing the final examinations of doctors to enable their early deployment into practice.^42^ On the other hand, following legal proceedings, the expansion of scope of practice of certain health occupations has been reversed in some cases on the grounds of patient safety.^43^

## Alignment with national goals

While health practitioner regulation is essential for identifying practitioners who have the minimum qualifications to provide safe care, there has been limited research on the links between health practitioner regulation and health system goals. The performance of regulatory systems is often limited to measurement of regulatory activities in isolation from patient outcomes. Evidence related to the effect of health practitioner regulation on population health outcomes is scare.^44^

There is also concern over regulation being used to reduce competition for patients, and to increase wages of incumbent practitioners by limiting entry into practice of newcomers to the regulated profession as well as new professions.^45^^,^^46^ Yet health workforce shortages and inequitable distribution of health professionals are major barriers to universal health coverage (UHC). Furthermore, regulatory systems that work in isolation and scattered across different occupations are reported to have constrained implementation of inter-professional education and team-based practice.^8^

Licensing – with restrictions placed on who can provide specific services or perform certain tasks – is recognized as the most intensive type of occupational regulation.^47^ Licensing can impose significant regulatory burdens and costs for the government, regulators, practitioners and the public, and is recommended only for occupations that pose the highest patient safety risk. Yet an increasing number of professions are pursuing this route. Some researchers have argued that licensing increases the labour supply.^48^ Other researchers, however, find that excessive restriction on education, scopes of practice and licensing can limit the supply and mobility of health practitioners. Such restrictions also increase the cost of health services and wages of practitioners, especially those who can work independently, without necessarily improving the quality of services.^45^^,^^46^^,^^49^^,^^50^

Because of the economic effects resulting from constraining the supply of health practitioners, regulation can be (mis)used to secure monopoly in the health labour market and protect the scope of practice of specific professional groups over evidence-based interventions to advance public welfare. There is concern that regulatory systems may be serving the interests of the professions (by constraining supply) and the regulators, rather than improving the quality of health professional education and practice.^23^^,^^49^

Nevertheless, there are important examples where health practitioner regulation has been used to strengthen health system goals. The conditional release of doctors’ professional licenses or certification on completion of rural service has helped place physicians in underserved areas in both high-income and low- and middle-income countries, for example in Australia, Ecuador, Nepal and Nigeria.^51^ In some settings, health practitioner regulation has been used to control entry to the private sector and minimize the negative consequences of dual practice (a phenomena in which a health practitioner is simultaneously employed in the public and private sector), for example in Botswana, Kenya, Myanmar and Uganda.^51^^,^^52^ Regulation has also been applied to control the financial costs incurred to students in health professions,^27^ inform health workforce planning and strengthen health-sector governance more broadly.^6^

There is need for greater research and policy attention on how to link health practitioner regulation to health system performance and patient outcomes to better serve the needs of health systems. Research on the effects of regulatory flexibility and innovations enacted in response to the COVID-19 pandemic should be considered to inform longer-term reforms.

## Conclusion

Important gaps exist between the potential of health practitioner regulatory systems and their actual contribution to UHC and health security. Common challenges include fragmented and ineffective regulatory systems; rigid education models; unnecessary requirements for entry to practice; restrictive scopes of practice; and reimbursement schemes that are occupation-specific. These issues may be impeding rather than supporting efforts to optimize the workforce, establish team-based practice and achieve equitable distribution of health workers, while increasing the cost of health services without any concomitant benefit to patients.

We need to critically examine how to best to align health practitioner regulation to a contemporary understanding of the public interest in specific contexts, including the prioritization of national health goals (Box 3). Regulatory systems are not static. Improvements are necessary to address the emerging needs for patient safety, and to incorporate advances in biomedicine and in information, communication and technology. Such improvements should be informed by regular review of regulatory policies and standards, not only on patient outcomes but also on health-system results.

Box 3 **Summary of key points in health practitioner regulation**

- There is substantial diversity in national health practitioner regulation systems based on underlying political, economic and social contexts.
- The primary objective of health practitioner regulation is increasingly expanding from patient safety to addressing the priorities of 21st century health systems.
- Gaps exist between the potential of regulatory systems and their actual use to support health-system goals.
- There are significant implementation challenges in low- and middle-income countries to regulation serving its intended functions.
- Regulatory adaptions during national emergencies present a strategic opportunity to reflect upon and better align regulatory systems to serve national health goals.

That said, the health practitioner regulatory reforms can only deliver results when the design is based on national needs, is appropriate to the context, balances multiple interests and has sufficient resources to operate effectively. The pandemic experience presents a unique opportunity to reflect on the priorities and objectives of health practitioner regulation, to generate evidence on health outcomes, and to prepare the health workforce and health system to be more responsive and resilient.
